# Group-level signatures in bonobo sociality

**DOI:** 10.1017/ehs.2024.44

**Published:** 2024-11-21

**Authors:** Edwin J. C. van Leeuwen, Nicky Staes, Marcel Eens, Jeroen M. G. Stevens

**Affiliations:** 1 Animal Behaviour and Cognition, Department of Biology, Utrecht University, Padualaan 8, 3584 CA Utrecht, The Netherlands; 2 Behavioural Ecology and Ecophysiology Group, Department of Biology, University of Antwerp, Universiteitsplein 1, 2610 Wilrijk, Belgium; 3 Centre for Research and Conservation, Royal Zoological Society of Antwerp, Koningin Astridplein 26, 2018, Antwerp, Belgium; 4 SALTO Agro-and Biotechnology, Odisee University of Applied Sciences, Hospitaalstraat 23, 9100 Sint Niklaas, Belgium

**Keywords:** Bonobos, sociality, intraspecific variation, group-level variation, *Pan paniscus*

## Abstract

Humans show remarkable differences in social behaviour between families, groups, communities and cultures, whereas such group-level within-species variation in socio-behavioural propensities is typically overlooked in other species. Studies on intraspecific variation in animal social structures are needed to inform an evolutionary account of human sociality. Here, we study multiple independent bonobo populations (*n* = 6) in zoological settings to investigate if and how bonobos (*n* = 70) show group-specific signatures in sociality. By applying tailored Bayesian statistical methods, we find that beyond individual and dyadic variation, the groups substantially differ from each other in core dimensions of great ape sociality: social proximity, grooming and play. Moreover, the groups’ network structures are distinct regarding cohesiveness and clustering, with some groups forming cohesive wholes, while others showcasing high levels of sub-grouping. Overall, while there is consistent evidence of differences in sociality between the groups, the patterns of cohesiveness and clustering are not consistent across the networks. This suggests that rather than groups having different levels of sociality, different patterns of sociality exist in each group. These findings warrant caution with characterising bonobos’ behavioural phenotype at the species level, and identify an essential source of variation that needs to be integrated in phylogenetic analyses.

## Graphical abstract

**Figure:**
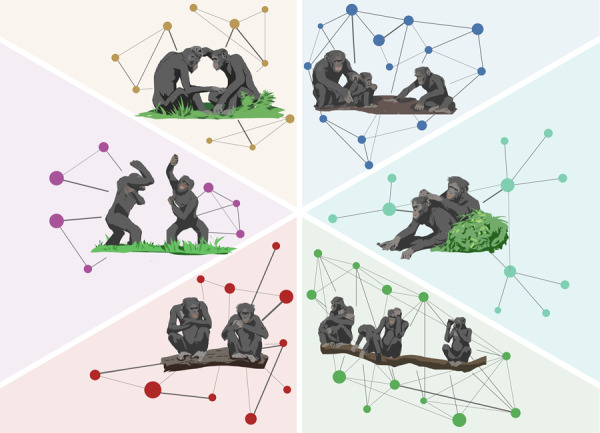

**Media summary**: Similar to human societies, bonobo groups show substantial diversity in their social behaviours, precluding broad species-level characterisations.

## Introduction

The human species is characterised by substantial behavioural variation at the individual (e.g. personality), dyadic (e.g. friendships) and group (i.e. cultures) levels. Such variability reflects phenotypic plasticity, which allows for a range of expressions that natural selection can act upon. A potent feature contributing to the biological success of the human species is its sociality, which can be defined as the tendency to associate and interact with others, forming social groups and networks. It encompasses a wide range of behaviours and structures that facilitate cooperation, communication and the establishment of social bonds (Bowles & Gintis, 2011; Enfield & Levinson, 2006; Tomasello, 2010). Whereas the study of human sociality traditionally focuses on (group-level) behavioural variation (Enfield & Levinson, 2006; McGrew, 1998; Mesoudi et al., 2006; Sperber, 1996), this focus is only marginally applied to studies of sociality in non-human animals (henceforth ‘animals’), where typically single populations are chosen to represent the entire species (Kaufhold & van Leeuwen, 2019; Lott, 1984; Strier, 2017; van de Waal, 2018). To gain more insight into the intricacies of animal behaviour and cognition, studies on (the range of) group-specific sociality are needed (Henrich & Boyd, 1998; Kendal et al., 2018; Schradin, 2013; Silk et al., 2009). For instance, only by comparing different groups of the same species may we be able to pinpoint whether culture has an influence on the respective species’ phenotypic expressions (Liebal & Haun, 2018; Nielsen & Haun, 2015).

Currently, there is compelling evidence that animals display substantial behavioural variation at the *individual* and *dyadic* levels. For instance, personality traits have been identified across various animal taxa (David et al., 2011; Kurvers et al., 2009; Massen & Koski, 2014; Tkaczynski et al., 2020; Verspeek et al., 2019; Webster et al., 2009), and long-lasting social bonds, which could be linked to ‘friendships’, are common among non-human primates (henceforth ‘primates’) (Berghänel et al., 2011; Mitani, 2009; Silk et al., 2003; Stevens et al., 2015). Such selectivity at the individual and dyadic level has been evidenced to affect fitness in animals (e.g. Silk, 2007; Webster et al., 2009), which speaks to the importance of studying behavioural variation in animal species. However, the study of behavioural variation becomes more challenging at the *group* level. Previous endeavours have started to detail aspects such as group-specific traditions (Langergraber et al., 2010; Luncz et al., 2012; Perry, 2011; Santorelli et al., 2011; van Leeuwen et al., 2012; Whiten et al., 2001) and variation in party size (Chapman et al., 1994; Furuichi, 2009), yet a clear focus on sociality is lacking and, moreover, comparisons are often confounded by the groups living in different habitats. In humans, variation in socio-cultural practices has been linked to influential behavioural dimensions like cooperation (Du et al., 2019; Henrich et al., 2005), fairness (Blake et al., 2015; Schäfer et al., 2015) and social learning (van Leeuwen, Cohen, et al., 2018). For instance, research on the social lives of South Indian Tamils revealed that post-marital residence patterns play a crucial role in shaping the kinship structures and social support networks, resulting in notable group differences in how these communities organise and sustain social support (Power & Ready, 2019). We envisage that also in animals, social network structures may afford or hamper opportunities for engaging in behaviours which could substantially impact fitness outcomes (Kaufhold & van Leeuwen, 2019; van de Waal, 2018).

Generally, group-level variation in sociality can be expected to arise based on ecological factors, genetics, demography and/or cultural variation. For instance, striped mice living in arid environments exhibit relatively abundant social lives, with several adults of both sexes sharing one nest. In contrast, when they live in moist grasslands, they live solitary lives, presumably owing to relative food scarcity (Schradin & Pillay, 2005). In primates, socio-ecological models have been used to explain effects of ecological conditions on group size and sociality (Clutton-Brock & Janson, 2012; Snaith & Chapman, 2007; Thierry, 2008; van Schaik, 1989). For instance, for macaques and chimpanzees, respectively, it has been found that in larger groups individuals are less socially connected (Balasubramaniam et al., 2018) and distribute their social resources (like grooming efforts) more selectively than in smaller groups (Escribano et al., 2022).

Such effects could be extended to other aspects of sociality like inter-individual distances (Enfield & Levinson, 2006; Hall, 1966), social cohesion (Gelfand et al., 2011; Uz, 2015) and inclinations to cooperate with one another (Henrich et al., 2005; House et al., 2020). These fundamentals of sociality are shaped by our everyday interactions within social circles, such as family, friends and living community, and form an integral part of our identities (Bornstein, 2012; Clegg & Legare, 2016; Enfield & Levinson, 2006; Keller et al., 2009; Tomasello, 2019; van Leeuwen, Cohen, et al., 2018). Moreover, they also determine the very opportunities we have to benefit from social life. For instance, the extent to which people interact with each other probabilistically influences their options to profit from social learning (Gelfand et al., 2011; Mesoudi et al., 2015; Richerson & Boyd, 2005; van Leeuwen, Cohen, et al., 2018). To date, however, there is limited knowledge on the extent of group-level variation in sociality in most animal species, including their possible consequences on individuals’ fitness (Kaufhold & van Leeuwen, 2019; van de Waal, 2018).

A few reports on animal behaviour allude to the significance of addressing such intraspecific group-level variation. For instance, observational studies on wild olive baboons revealed a remarkable phenomenon wherein a troop maintained a culture of ‘pacifism’ despite significant demographic changes (Sapolsky & Share, 2004). Even after the deaths of aggressive males owing to tuberculosis, the troop sustained peaceful interactions characterised by high rates of grooming, relaxed dominance hierarchies and non-aggressive behaviours between resident females and new immigrants (Sapolsky, 2006). Furthermore, translocation studies with macaques highlighted the adaptability of social behaviour in response to exposure to different socio-behavioural patterns. Juvenile rhesus macaques exhibited increased reconciliation rates after being housed with stumptail macaques, indicating a process of cultural assimilation where individuals adopt behaviours prevalent in the new social environment (de Waal & Johanowicz, 1993). Albeit rare, also among non-primate species group-specific sociality has been documented. Research on sperm whale clans illustrated the diversity in social behaviours among distinct groups. Each clan exhibited unique social dynamics, indicating that sociality is not homogenous within a species and can vary significantly between different groups (Cantor & Whitehead, 2013, 2015). Specific to the great apes, regional variation in social behaviour has been reported in the wild. Studies on chimpanzee populations revealed regional differences in social behaviour, with west-African female chimpanzees exhibiting higher levels of sociality compared with their east-African counterparts. However, recent research suggests that nuances in group identity rather than subspecies level might play a significant role in shaping social dynamics (Koops et al., 2024; Lehmann & Boesch, 2008).

To understand the range of intraspecific group-level variation in our closest living relative – the bonobo (*Pan paniscus*) – in the current study, we adopted a multiple group approach using a standardised observation protocol to quantify individual, dyadic and group-level rates of social interactions. Specifically, we investigated whether zoo-housed bonobos exhibit group-level variation in three core dimensions of sociality: keeping proximity (close and distant), grooming and play. We operationalise sociality in terms of the frequencies of daily modes of interacting and explicitly focus on group-level variation as an overlooked source of variation in animal sociality (Kaufhold & van Leeuwen, 2019; Lott, 1984; Strier, 2017; van de Waal, 2018). In this realm, in bonobos, there are a few reports on group differences in traditions (Hohmann & Fruth, 2003; Samuni et al., 2020; van Leeuwen et al., 2020), yet systematic investigations of intraspecific variation in their general (group-)levels of sociality are lacking. We studied six groups of bonobos in similar environmental conditions using the same methodological approach. Recently, a study in three groups of wild vervet monkeys found that the extent to which the vervets engaged in affiliative behaviour (e.g. social proximity, grooming) was dependent on which group they lived in, and that these group-specific patterns were relatively stable over a 9 year period (Kerjean et al., 2023). In the current study, we developed a tailored Bayesian approach to quantify variation in sociality at the individual (Verspeek et al., 2019; Weiss et al., 2015), dyadic (Stevens et al., 2015) and group level, while controlling for temporal autocorrelation and demographic variation at the group level (i.e. group size and sex ratio). To further quantify sociality beyond individuals and dyads, we derived social network metrics from the four respective sociality variables to test the hypothesis that bonobos’ sociality is dependent on group identity. This novel quantification of bonobo sociality holds the potential to identify a hitherto overlooked dimension of their (group-specific) social lives and highlight a valuable component for empirical scrutiny in future comparative studies on our closest living evolutionary relatives (van Leeuwen et al., 2023).

## Methods

### Procedure

We studied six zoo-housed groups of bonobos to determine whether group signatures of sociality can be identified over and above individual- and dyad-level variation. As measures of sociality, we focused on socio-positive behaviours pivotal to bonobo societies: close proximity (0–1 m), distant proximity (1–2 m), social play and allogrooming (Furuichi, 2019).

### Subjects

The study sample comprised 70 bonobos (of which 28 males) across six independent zoo-housed populations. We focused on individuals aged 7 years or older (*n* = 50), because of their relative independence with respect to making social choices. Previous research on captive bonobos suggests that the onset of puberty is likely to occur from approximately 6 to 10 years of age, with the sharpest increase in urinary testosterone around 8–9 years of age for males and an earlier but more gradual increase in females (Behringer et al., 2014). Given uncertainty for individual subjects we decided to opt for a wide range and included all individuals aged 7 years and older into our analysis. The individuals younger than 7 years old were included as potential recipients of social behaviour, but not analysed as main actors (i.e. focal subjects). This means that all social behaviours of the focal subjects were included in this study, whether they were directed to other adolescents/adults or to individuals younger than 7 years old. Focal subjects’ mean age ± SD = 21.7 ± 12.7 years (age range 7–63 years). Group sizes ranged from eight to 16 individuals (which we accounted for in the analysis; see the section ‘Data analysis’). Table S1 provides all subject information including group identity, sex, and age at the start of the study.

All bonobos were housed adherent to the guidelines of the European Association of Zoos and Aquaria (EAZA) Ex-situ Program (Stevens, 2020). Bonobos had access to an indoor and outdoor enclosure at all zoos, with the exception of Wuppertal, which only had indoor enclosures at the time. Indoor and outdoor areas always contained large permanent climbing structures and nesting platforms. All groups had unlimited access to drinking water, were provisioned by keepers two to five times a day and were provided with similar amounts of enrichment throughout the period of observation (although this was not quantified in detail). Importantly, we note that even in living quarters much smaller than the wild habitats, great apes selectively choose who to associate and interact with (Kanngiesser et al., 2011; van Leeuwen et al., 2019). This means that obtained measures of sociality reflect the apes’ social decisions rather than forced choices.

### Data collection

Data were collected with standardised protocols consisting of scan sampling at the onset of 10 min focal follow sessions. The scan sampling procedure yielded point behaviours and consisted of a group scan in which we scored social behaviours for all visible subjects, including their respective partners. We opted to only use these scan data to minimise data dependency (which is much higher during continuous data scoring in focal follows) (Martin & Bateson, 2007; Whitehead, 2008). *Close proximity* was defined as being within 1 m distance or in physical contact without further interacting, *distant proximity* was defined as being within 2 m distance without further interacting (mutually exclusive with *close proximity*), *play* was defined as two or more individuals engaging in one or more activities such as tickling, gentle grabbing, pirouetting, pulling and/or pushing, slapping, sliding, and rough and tumble (Palagi, 2006), and *grooming* was defined as fur cleaning performed by one individual on another via hands or mouth (Sakamaki, 2013). If subjects were not interacting, the scan counted towards non-sociality in the analyses; if subjects were out-of-sight (yet present in their enclosure), they were only included in relation to subjects who were in sight, as this logically means that the non-visible subjects were not interacting with the visible ones (see the Supplementary Information). Data were collected by six observers under supervision of one of the co-authors (NS). Each observer received 3 weeks of training prior to data collection. After each student was trained, their inter-observer reliability was tested by scoring two 10 min bonobo focal video recordings. High Spearman rank correlations (mean *ρ* ± SD = 0.86 ± 0.15) were found across all observers coding the behaviours (Martin & Bateson, 2007b). Each group was daily sampled for a 3 month period between 2012 and 2014. A total of 5232 scans (leading to *n* = 35,939 data points) were obtained across the six groups (range 521–1286 scans). Our Bayesian data analyses incorporated measures of uncertainty accounting for the variation in sampling effort.

### Data analysis

#### a. Quantifying sources of variation for different interaction types

We used a Bayesian generalised linear mixed model to assess variation between and within groups in the extent to which bonobo dyads (1) spent time in proximity, (2) engaged in play together and (3) groomed one another. The analysis of time spent in proximity was repeated based on two criteria differing in intensity: (1a) *close proximity* (within 1 m distance, at rest, not interacting); and (b) *distant proximity* (within 2 m distance, at rest, not interacting). We distinguished between these two measures of proximity because in the former one could engage physically with the partner, whereas the latter precludes this (yet still reflects a level of nearness). In all cases a binary response variable of the form *y*_*ijklm*_ represents whether individuals *i* and *j* in group *k* (henceforth ‘dyad *ijk*’) were recorded as engaging in the target interaction (henceforth ‘interacting’) on scan *l* on day *m* (1) or not (0). We modelled this using a logit link function and Bernoulli error structure. Thus, the probability that dyad *ijk* was interacting for a given scan *P*(*y*_*ijklm*_ = 1) was modelled as follows:1
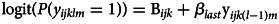


where y_*ijk*(*l*−1)*m*_ indicates whether dyad *ijk* was interacting during the previous scan (*l −* 1), set to zero for the first scan of each day. Therefore *β*_*last*_ is a parameter allowing for any tendency for dyads to engage in bouts of interactions that continue across scans within a day, and thus allows for the resulting non-independence of successive scans. B_*ijk*_ is then a linear predictor of random effects measuring the propensity for members of dyad *ijk* to engage in the target interaction. For *close proximity, distant proximity* and *playing*, this was defined as:2
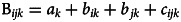
where *a*_*k*_ is a random effect of ‘group’, distributed as 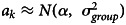, *b*_*ik*_ is a random effect of ‘individual’, distributed as 
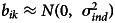
 and *c*_*ijk*_ is a random effect of ‘dyad’, distributed as 
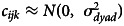
. The parameter *α* estimates the overall population mean (on the log odds scale). Variation between dyads in the tendency to interact is broken down into three sources of variation: group level, individual level and dyad level, with the relative magnitude of each being estimated by the standard deviation (SD) of each effect, respectively *σ*_*group*_, *σ*_*ind*_, *σ*_*dyad*_. The estimated relative magnitude of these effects tells us the extent to which groups vary in their propensity to interact relative to the variation within groups. The overall SD within groups can be calculated as 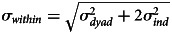, and we can then quantify the ratio of SD between/within groups as *σ*_*group*_/*σ*_*within*_.

Furthermore,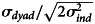 provides a measurement of how dyads vary within groups; 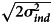 is used instead of *σ*_ind_ since this component of variance is counted twice – via *b*_*ik*_ and via *b*_*jk*_. We denote this measure as *σ*_*dyad*_/*σ*_*IND*_ where *σ*_*IND*_ is thus the standard deviation of the variance component accounted for by individual tendencies. For example, if, at one extreme, bonobos vary in the extent to which they are in proximity to others, but each has no preference in whom they are in proximity with, we would expect *σ*_*IND*_ to be large relative to *σ*_*dyad*_. If, on the other hand, bonobos differ in their preferences for who they want to be in proximity with, we would expect *σ*_*dyad*_ to be large relative to *σ*_*IND*_.

Since *grooming* is a directional interaction, one individual was recorded as the groomer and the other as the groomed (recipient), which leads to the inclusion of one additional random effect for *grooming*:3
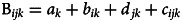
Here *b*_*ik*_ is now individual *i*'s propensity to groom and *d*_*jk*_ is individual *j*'s propensity to be groomed, with standard deviation *σ*_*rec*_ (rec = recipient). Consequently, for grooming, we have:
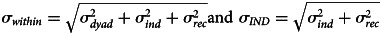
Bayesian estimation was accomplished using Markov Chain Monte Carlo methods using the JAGS (Plummer, 2003) sampler, via the runjags (Denwood, 2016) and coda (Plummer et al., 2006) packages in the R statistical environment. Vague (uninformative) priors were specified for all model parameters, with *α*, *β*_*last*_ ≈ *N*(0, 10000) and *σ*_*group*_, *σ*_*ind*_, *σ*_*dyad*_ ≈ *U*(0, 10). Further descriptions of all models and the estimation procedure are provided in the Supplementary Information.

#### b. Estimation of social networks and network metrics

We also sampled posterior distributions for B_*ijk*_ in each case, which enabled us to derive posterior distributions for the social networks representing the proportion of time engaged in each interaction for each dyad in each group (see the Supplementary Information). While *playing* and *grooming* are types of interaction, for which networks with rates of interaction are typically used, the scan sampling nature of our data makes an association-type network most appropriate. For example, *n*_*groom*,*ijk*_ estimates the probability that *i* will be grooming *j* in group *k* at a random point in time, as opposed to quantifying the rate at which *i* initiates grooming of *k* per unit time. Our Bayesian modelling approach has a number of advantages over calculating a single number for each connection using e.g. the simple ratio index (Whitehead, 2008). First, it controls for autocorrelation in successive scans. Second, it provides a measure of uncertainty in the value of each connection, which also allows us to quantify the uncertainty in metrics derived from them, simply by calculating metrics for each iteration of the Markov Chain Monte Carlo, thus obtaining a posterior sample (see the Supplementary Information).

Furthermore, we derived two node-based metrics for each individual: (1) strength – total connection to others in the group, e.g. 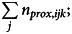 and (2) cluster coefficient using the approach of Ahnert et al. (2007) (see the Supplementary Information). For each iteration we then calculated the average strength and clustering across the subjects in each group enabling us to assess whether groups differed in these measures. Thus, we obtained posterior samples for network metrics at individual and group levels enabling us to derive estimates (mean of posterior) and 95% highest posterior density (HPD) intervals. We were also able to obtain 95% HPD intervals for the difference in strength and clustering between each pair of groups and test for evidence of a difference between each pair of groups (Tables S3–S10). Lastly, we tested whether groups may differ in their extent to which they form *cliques* of interacting individuals (Girard-Buttoz et al., 2020; Sakamaki, 2013) (e.g. more than two individuals at the same time), over and above that predicted by the dyadic interaction rates estimated in the model. Overall, we inferred evidence for group-level variation in sociality when the 95% highest posterior density interval (HPDI) for the difference between groups did not include zero.

#### c. Effect of dyad-level variables

We expanded the models described in Eqns (1)–(3) to include dyad-level variables in the linear predictor B_*ijk*_ (see the Supplementary Information for model definitions). For non-directional interactions (close proximity, distant proximity and playing) we included the magnitude of the difference in age (standardised across all groups), two binary variables indicating whether a dyad was maternal and paternal kin, and whether they were a different sex or both male (with female dyads set to the reference level). For grooming (directional) age difference was entered as age of groomer – age of groomed (standardised across all groups). There were also two indicator variables for mixed sex dyads: males grooming females and females grooming males. Group-level variables were also considered, but with only six groups we had insufficient statistical power to draw any conclusions (see the Supplementary Information).

## Results

### Descriptive statistics

For each dyad, and each of the four sociality metrics, we calculated the ‘raw’ proportion of scans in which the target behaviour was occurring. We then summed across dyads to get a total proportion of time engaged in the activity for each individual. Afterward, we took the mean and standard deviation across groups (Table 1). Here, we can already see substantial differences across the groups, which we test more formally in the sections below.

**Table 1.** Mean (*μ*) and standard deviation (*σ*) of the dyadic association and interaction rates in the six sampled groups of bonobos

	Close proximity μ *σ*		Distant proximity μ *σ*		Play μ *σ*		Grooming μ *σ*	
Apenheul	0.626	0.232	0.038	0.022	0.024	0.014	0.103	0.05
Frankfurt	0.396	0.162	0.149	0.064	0.029	0.034	0.118	0.057
Planckendael	0.604	0.171	0.207	0.101	0.012	0.019	0.103	0.05
Stuttgart	0.519	0.174	0.231	0.107	0.017	0.023	0.066	0.039
Twycross	0.386	0.165	0.168	0.076	0.021	0.016	0.107	0.045
Wuppertal	0.465	0.175	0.229	0.042	0.033	0.034	0.114	0.051

To formally test whether the groups of bonobos differed from each other in their levels of sociality, we computed the independent contributions of individual, dyadic and group-level variation to test the hypothesis that comparable groups of bonobos differ from one another in terms of core social dimensions even after accounting for expected sources of variation in sociality (including temporal dependency; Whitehead, 2008). Furthermore, we computed two common group-level social metrics (i.e. average strength *and* clustering; Sosa et al., 2021; Whitehead, 2008) to enable comparison of sociality at the group-level. Lastly, we quantified sociality at the group-level by investigating the occurrences of cliques of individuals associating or interacting at the same time (Girard-Buttoz et al., 2020; Sakamaki, 2013).

### Group-level signatures

**Figure 1.**
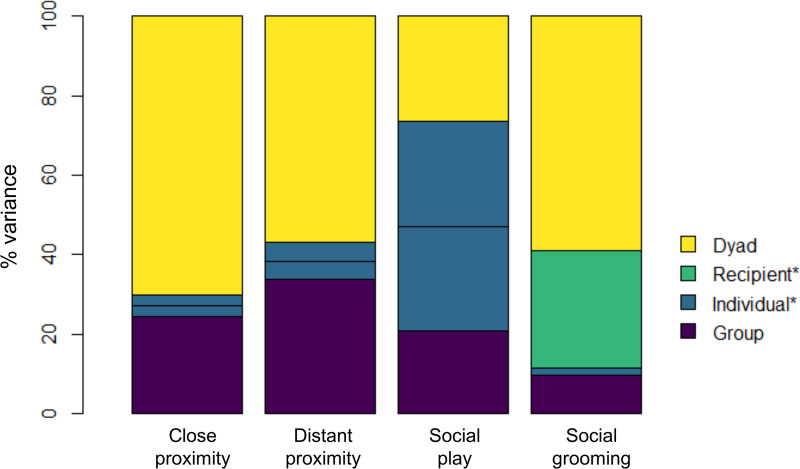
Breakdown of variance in the log-odds of interaction across all dyads for four interaction types. *Individual variance counts twice towards the overall variance for non-directional interactions. In contrast, for grooming (directional), there is a separate component for variation in the extent to which individuals groomed others (individual) and to which they were themselves groomed by others (recipient).

In all four analyses, the group level standard deviation (*σ*_*group*_) estimated with 95% HPDIs is clearly away from zero (Table 2), indicating that there is strong evidence of systematic differences between groups in sociality that are not fully accounted for by sampling error at the level of individuals and dyads. In other words, it is statistically unlikely that the observed differences in sociality between the study groups are purely a result of differences in the sociality of individuals and dyads that comprise those groups. In all four network types, the between-group variation is estimated to be smaller than the within-group variation, but of a plausibly similar magnitude for both proximities and play with 95% HPD intervals for *σ*_*group*_/*σ*_*within*_ including 1. Table 2 shows the estimates of the standard deviations for each component of variance (see Table S2 for the estimated population means for each behaviour), while Figure 1 shows the components broken down as an estimated percentage of variance.

**Table 2.** Summary of posterior estimates for random effects in all four analyses. The top three rows give the mean of the posterior sample for the group, dyad, and individual level random effects, respectively, the fourth row for the recipient of grooming. The fifth row gives the mean of the posterior sample for *σ*_*group*_/*σ*_*within*_ , providing an estimate of the relative between and within group variation. The sixth row gives the mean of the posterior sample for *σ*_*dyad*_/*σ*_*IND*_ providing an estimate of the relative importance of dyad- vs. individual-level variation. The 95% highest posterior density intervals (HPDIs) are given in brackets. Shaded cells indicate that the 95% HPD interval for ratio of two SDs does not include 1, meaning that either the numerator or denominator receives more weight. In that case, we infer evidence for one of the sources of variance being more influential than the other

	Close proximity	Distant proximity	Play	Grooming
*σ*_*group*_	0.850 (0.071,1.843)	1.056 (0.342,2.125)	1.672 (0.505,3.466)	0.746 (0.157,1.52603)
*σ*_*dyad*_	1.413 (1.207,1.625)	1.291 (1.115,1.481)	1.857 (1.337,2.440)	1.942 (1.730,2.152)
*σ*_*ind*_	0.246 (0.000,0.488)	0.356 (0.069,0.605)	1.884 (1.449,2.301)	0.293 (0.000,0.601)
*σ*_*rec*_				1.378 (0.959,1.794)
*σ*_*group*_/*σ*_*within*_	0.582 (0.0403,1.260)	0.758 (0.222,1.516)	0.515 (0.151,1.058)	0.309 (0.0632,0.646)
*σ*_*dyad*_/*σ*_*IND*_	20.287 (1.176,41.453)	5.172 (1.129,8.119)	0.712 (0.473,0.988)	1.399 (0.980,1.867)

For *close proximity* and *distant proximity*, the dyad-level variation dominates the individual-level variation (Table 2; Figure 1), suggesting that, within groups, individuals tend to be similar in their propensity to be in proximity to others, but that they have strong preferences in whom they associate with. In the *play* analysis, the individual variation is estimated to be slightly greater than the dyad-level variation (Figure 1), with *σ*_*dyad*_/*σ*_*ind*_ estimated at 0.712 with HPD interval 0.473–0.988 (Table 2), indicating that individuals vary substantially in their playfulness, but that each also has favoured playmates. Finally, variation between recipients of *grooming* is much greater than between groomers (Table 2; Figure 1), indicating that there are certain individuals who tend to be groomed a lot and individuals who tend not to be groomed (more so than the difference between the extent to which individuals groom others).

Importantly, all the reported outcomes exist while controlling for the significant effect of autocorrelation between successive scan points (see Supplementary Tables S2, S11 and S12) and for the effects of group size and sex ratio. These latter two group-level effects, however, could not be estimated with any reasonable level of certainty owing to low power at the group level (see the Supplementary Information Section S3).

### Social network metrics

**Figure 2.**
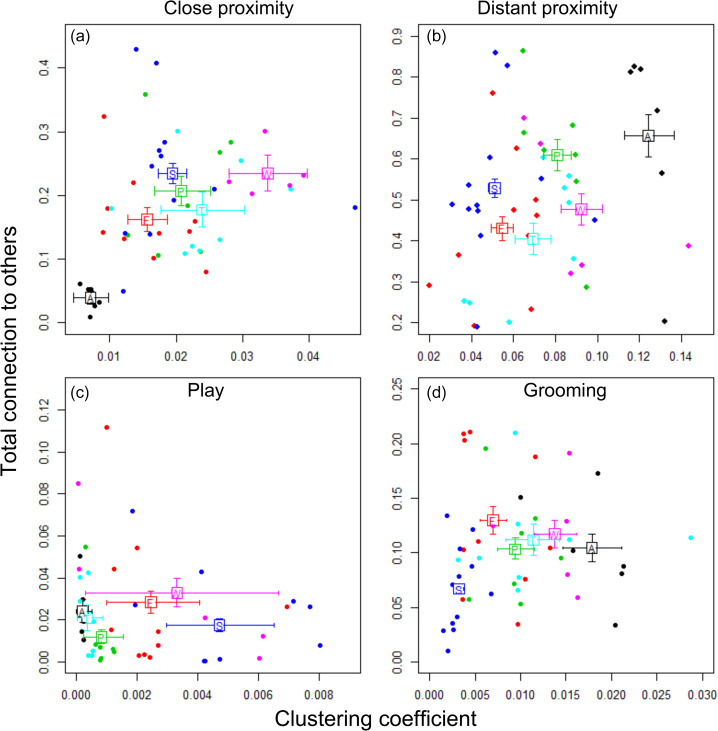
Group differences in the node-based measures *strength* (total connection to others) and *clustering coefficient* (the extent to which the neighbours of one individual tend to be linked to one another). The coloured squares are the estimates (mean of posterior distribution) of group means across subjects: A, Apenheul; F, Frankfurt; P, Planckendael; S, Stuttgart; T, Twycross; W, Wuppertal. Error bars show 95% highest posterior density intervals. Points represent individuals with colours indicating group membership.

For *close proximity*, *distant proximity* and *play*, the six bonobo groups clearly differ from one another in terms of estimated average strength of sociality (total connection) and estimated average clustering coefficient (see Tables S3–S10 for the group contrasts and Figure 2 for the overall estimates). In contrast, for *grooming*, most groups appear similar in average strength, except for the Stuttgart group, for which average strength is considerably lower (Table S9). For clustering of grooming interactions, again, the groups differ substantially from each other (Table S10; Figure 2d).

Furthermore, we investigated a measure of extended dyadic interactions, namely the extent to which groups form *cliques* of interacting individuals (Sakamaki, 2013). Here, for all measures except for grooming, we found substantial differences between groups over and above that expected by the dyadic interaction rates (see Section S6). Lastly, we aimed to check whether the observed group differences in sociality may be specifically influenced by group size and/or sex ratio. However, owing to a lack of power, the models were unable to identify whether any of such effects were present in the data (see Section S3).

In summary, while there is consistent evidence of differences in sociality between groups, the patterns of strength and clustering are not consistent across the four networks. This suggests that rather than groups having different levels of sociality, different patterns of sociality exist in each group, e.g. some groups groom more whereas other groups play more, or are more likely to associate in (close) proximity.

### Effects of dyad-level variables

We found evidence that groups differed in their rates of association (close proximity and distant proximity) and interaction (play and grooming) and that the variation within groups was predominantly at the dyad, rather than the individual level. Therefore, we next assessed what variables might drive such differences between and within groups (for model specifications, see the Supplementary Information).

#### Kin effects

For all four sociality measures, there is strong evidence that maternal kin are much more likely to interact than other dyads. Back transforming to the odds scale, at a random point in time, maternal kin are an estimated 6.1× (95% HPDI: 2.7–14.3) more likely to be *in close proximity*, 3.5× (1.6–7.8) more likely to be in *distant proximity*, 12.2× (5.1–31.5) more likely to be *playing* together and 15.8× (7.8–31.2) more likely to be *grooming* one another than dyads who are not maternal kin. There is little evidence that this effect also applies to paternal kin, except for *play* where paternal kin dyads are an estimated 1.49× (1.1–17.0) more likely to be engaged in play than dyads who are not paternal kin. Estimates of all parameters with highest posterior density intervals can be found in the Supplementary Information.

#### Sex effects

There is strong evidence that female dyads spend more time in *close proximity* and *distant proximity* than other sex combinations, although this effect is weaker than the effect of maternal kinship. Back transforming to the odds scale, at a random point in time female dyads are an estimated 2.0× (95% HPDI: 1.3–3.2) more likely to be in *close proximity* and 1.8× (1.6–3.0) more likely to be in *distant proximity* than mixed sex dyads. Female dyads are also an estimated 2.9× (95% HPDI: 1.2–6.9) more likely to be in *close proximity* and 2.8× (1.2–6.6) more likely to be in *distant proximity* than male dyads. There is little evidence of sex effects on *play* or *grooming*, nor of any difference between male dyads and mixed sex dyads in any analyses. Likewise, there is no evidence that males groom females more than females groom males or vice versa. However, the 95% HPD intervals are wide in all cases (see the Supplementary Information), so sizeable effects in either direction cannot be ruled out based on these data.

#### Age effects

There is reasonable evidence that dyads more similar in age spend more time in *proximity* than dyads less similar in age. Back transforming to the odds scale, an increase of 1 SD in age difference leads to a predicted 0.75× (95% HPDI: 0.58–0.98) reduction in the time spent in *close proximity*. The effect goes in the same direction for *distant proximity*, but the 95% HPDI includes zero indicating a lack of strong evidence. There is little indication of an age effect on *play* or on *grooming*.

## Discussion

While controlling for individual and dyadic variation between bonobos, in this study, we show that groups of bonobos have their own group-specific sociality, where some groups engage more in grooming, while other groups express their sociality more in terms of social proximity without interaction. Furthermore, some groups of bonobos were much more tightly connected (cf. social network strength and clustering) and engaged in larger-group social congregations (i.e. cliques) than others, which indicates that within the bonobo species, substantial group-level variation exists with respect to the expression of their social phenotype. In this light, we find support for our hypothesis that bonobos’ sociality is dependent upon group identity (cf. Kerjean et al., 2023) which implies that species-level typologies like ‘peaceful bonobos’ or ‘aggressive chimpanzees’ (Furuichi, 2011; Gruber & Clay, 2016; Nurmi et al., 2018; Parish, 1994) lose credibility (also see van Leeuwen et al., 2023).

For all the reasons that (close) proximity and grooming are important in the lives of bonobos (Allanic et al., 2020; Dunbar, 1991; Furuichi, 2019; Sakamaki, 2013; Samuni & Surbeck, 2023), our findings mean that bonobos in some groups may experience more favourable social conditions than in others. In other words, their group-level sociality could correlate with individuals’ potential for adaptive interactions. Under wild scenarios, this could play out in differential fitness outcomes, which reiterates the importance of studying group-level variation in sociality. For instance, innovations such as foraging skills, or predator avoidance strategies spread faster in tightly knit societies (Barkoczi & Galesic, 2016; van Boekholt et al., 2021) and certain behaviours are transmitted with differential efficiencies based on the type of networks involved (e.g. grooming vs. proximity networks) (Boogert et al., 2014; Hasenjager et al., 2020; van Leeuwen et al., 2020; Voelkl & Noë, 2008). Similarly, variations in network structures may selectively promote cooperative interactions, potentially impacting individuals’ competitive advantages (Apicella et al., 2012; Voelkl & Kasper, 2009). As such, taking intraspecific group-level variation seriously when studying the adaptive value of behavioural traits seems essential (Kaufhold & van Leeuwen, 2019; van de Waal, 2018).

In more detail, we observed that maternal kin biases social behaviour across all metrics, while sex only affects proximity, with females being more likely to stay close together compared with other sex combinations. Interestingly, this effect was not observed for grooming or playing. Additionally, bonobos of similar age tend to stay in proximity more than those with larger age differences, indicating a cohort effect, although this trend did not apply to grooming or playing. These findings are consistent with earlier reports on bonobos’ social life (Furuichi, 2011, 2019; Parish & De Waal, 2000), and beg the question to what extent family ties and sex ratios affect group-level sociality. In the current study, we tested the effects of group size and sex ratio on the bonobos’ sociality, yet, owing to a relatively small sample size at the group level (*N* = 6), these analyses yielded large magnitudes of uncertainty, leaving the question open for future research incorporating more groups (Torfs et al., 2023; van Leeuwen et al., 2023).

The use of a standardised data-collection procedure across groups and the tailored statistical approach of separating individual, dyadic and group-level variation yield a novel quantification of bonobo sociality across groups and ensure that our findings have a number of plausible implications.

First, the assumption that studying one group of animals of a given species justifies generalisations at the species level is rendered questionable, at least for spontaneously expressed social behaviour in great apes (Cronin et al., 2014; Kaigaishi et al., 2019; van Leeuwen et al., 2021, 2023). An earlier study already reported that bonobos’ flagship trait of female bonding is not universal across different groups of bonobos (Stevens et al., 2006) and recent findings in four groups of sanctuary-housed chimpanzees have similarly identified substantial group-level differences in social behaviour (van Leeuwen, Cronin, et al., 2018). This implication may be especially relevant for attempts to trace the phylogenetic origins of behaviours, which seems daunting in the absence of knowledge on the breadth of variation in the species under study (Garamszegi, 2014; Kamilar & Cooper, 2013; Strier, 2017). Phylogenetic regression analyses aim to trace the evolutionary paths of behaviours and uncover mechanisms behind specific behavioural outcomes (Lycett et al., 2007; MacLean et al., 2012). These analyses focus on species-typical behaviours, which raises the question: ‘what is species-typical behaviour?’ Behaviour evolves more flexibly than morphology or physiology (Blomberg et al., 2003), making it difficult to estimate behavioural phenotypes accurately. Intraspecific variation further complicates phylogenetic analyses by adding another layer of variability. To address this, it is suggested to estimate parameter values by weighing species’ influence based on the number of sampled groups and subjects per group. Methodologically, however, increasing sample sizes – particularly the number of groups per species – and the sampling effort within groups are crucial.

Second, our findings corroborate the observation that bonobos exhibit a high level of behavioural flexibility (Hohmann & Fruth, 2003), which informs the debate about the evolution of adaptive potential in humans and other hominins (de Waal, 1994; Stevens et al., 2008; van Schaik, 2013). The adaptive potential of humans refers to our ability to adjust and thrive in various environments and situations. This adaptability is rooted in our cognitive abilities, social structures and behavioural flexibility, e.g. we can learn from experiences, innovate and collaborate to solve problems, which has been crucial to our survival and success as a species (Henrich, 2016; Tomasello, 2014). Apparently, bonobos are highly flexible animals too, in the sense that their most fundamental social behaviour encompasses substantial degrees of freedom to attune to local circumstances (Stevens et al., 2008). In conjunction with similar positive evidence from humans’ other closest living relative – the chimpanzee (de Waal, 1994) – this indicates that the phylogenetic appearance of such adaptive potential predated the emergence of the hominin lineage.

Third, our results are consistent with an explanation in terms of social learning, which leads to the final implication of our findings: bonobos’ social lives may be (partly) shaped by cultural processes. Bonobos are known to engage in cultural behaviours (Hohmann & Fruth, 2003; Samuni et al., 2020; van Leeuwen et al., 2020), but to date, such characterisations of culture have mostly been limited to isolated and clearly delineated behavioural forms, like hunting preferences (Samuni et al., 2020) or groom-slapping (van Leeuwen et al., 2020). Our current results are consistent with the possibility that bonobos’ social networks can themselves be culturally governed. For instance, bonobos may learn from observing their group members which social behaviours are part of their group's preferred behavioural repertoire, or even which behaviours are most conducive to favourable outcomes (e.g. eliciting affiliative responses). Such social learning does not have to be cognitively demanding or comprise detailed know-how copying (Tennie et al., 2020), yet could nonetheless result in the within-group behavioural synchrony observed in this study (e.g. see (Berthier & Semple, 2018; Koski & Burkart, 2015; Ostner et al., 2021)). Such cultural shaping of sociality may go hand in hand with genetic (Staes et al., 2014) and demographic (DeTroy et al., 2021) influences (Koops et al., 2014; Schradin, 2013), yet warrants closer scrutiny given its hitherto overlooked status in the study of animal (but not human) culture.

An alternative driver of variation in bonobo sociality in zoological settings (this study) may be the differential housing conditions in terms of e.g. enclosure space, diet, and husbandry style. We did not conduct detailed analyses on such parameters because research has shown that great apes, even in confined spaces like zoos, deliberately choose their interaction partners, especially for close proximity (0–1 m) and grooming (Kanngiesser et al., 2011; Stevens et al., 2008; van Leeuwen et al., 2019). Moreover, there is a wealth of studies showing that apes form long-lasting social relationships, both in the wild and in zoological settings, which further points towards them selectively seeking out individuals to associate and/or interact with (Massen & Koski, 2014; Stevens et al., 2006; Verspeek et al., 2019). The impact of other factors like husbandry style, diet and/or enrichment programmes may be charted in future studies, but at this point, there is reason to assume that these factors are negligible in explaining the group differences we observed in the current study, as all zoos are EAZA accredited and have as such similarly high standards with respect to ape management.

Furthermore, we acknowledge that our data-collection windows encompass only a fraction of the lifespan of the apes, and that longitudinal data would be valuable to pinpoint determinants of sociality and possibly to identify regulating mechanisms for maintaining group stability (e.g. policing (Flack et al., 2006) and conformity (Tkaczynski et al., 2020; van Leeuwen, 2021)). Nonetheless, this cross-sectional approach exposes variation between the ape groups that could affect their respective responses to all kinds of (experimental) conditions, like visitor effects (Davey, 2007), cooperation opportunities (Suchak et al., 2016; van Leeuwen et al., 2021) and unequal reward distributions (Brosnan et al., 2005). For this reason alone, future studies would benefit from a multi-group approach or even from regressing socio-demographic variation on the response variable under study to chart its reaction norm (e.g. see DeTroy et al., 2021). Finally, it remains important to test to what extent the findings of these zoo-housed populations hold for bonobos (or any other socially living species) in the wild (e.g. see Koops et al., 2024 for an indication in chimpanzees).

In summary, the current study provides important evidence that – beyond the hitherto reported individual (Verspeek et al., 2019) and dyadic variation (Stevens et al., 2015) in social behaviour – groups of bonobos can substantially differ from one another in fundamental domains of their social lives. A relevant next step would be to investigate if groups of bonobos, contingent on their (current) group-level sociality, may also respond differently to situations requiring social cooperation, such as sharing resources or coordinating during group activities (Samuni et al., 2021; Samuni & Surbeck, 2023; van Leeuwen et al., 2021). Such investigations could ground the observed group-level variation into an adaptive evolutionary framework.

## Supporting information

van Leeuwen et al. supplementary material van Leeuwen et al. supplementary material

## Data Availability

All data used in this study are available at the following public repository: https://surfdrive.surf.nl/files/index.php/s/9FM1BQfzcHhF0R4

## Supplementary material

The supplementary material for this article can be found at https://doi.org/10.1017/ehs.2024.44.

## Code availability statement

The code used in this study is available upon reasonable request from the first author (Edwin J. C. van Leeuwen).
