# Supplementary material for: Group-level signatures in bonobo sociality
Source: Evol Hum Sci. 2024 Nov 21;6:e48. doi: 10.1017/ehs.2024.44 (PMC11660378; doi:10.1017/ehs.2024.44)
Supplement: van Leeuwen et al. supplementary material [file S2513843X24000446sup001.docx]

**Supplementary Information for:**

**Group-level signatures in bonobo sociality**

Edwin J. C. van Leeuwen^1-3^, Nicky Staes^2,3^, Marcel Eens^2^, Jeroen M. G. Stevens^4^

^1^ Animal Behaviour and Cognition, Department of Biology, Utrecht University, Padualaan 8, 3584 CA Utrecht, The Netherlands

^2^ Behavioural Ecology and Ecophysiology Group, Department of Biology, University of Antwerp, Universiteitsplein 1, 2610 Wilrijk, Belgium

^3^ Centre for Research and Conservation, Royal Zoological Society of Antwerp, Koningin Astridplein 26, 2018, Antwerp, Belgium

^4^ SALTO Agro- and Biotechnology, Odisee University College, Hospitaalstraat 23, 9100 Sint Niklaas Belgium

**Methods**

**S1. Quantifying sources of variation for different interaction types**

We used a Bayesian generalized linear mixed model (GLMM) approach to assess variation within and between groups in the extent to which individual bonobos, *i*) associated with each other (divided in 2 measures): close proximity (physical contact or <1m distance) and distant proximity (1-2m)); *ii*) engaged in social play together (henceforth *play*); and *iii*) groomed one another. Here, we first describe the model used in the context of the proximity analysis (1a).

***S1.1 Proximity model***

The binary response variable$t_{ijklm}$ represents whether individuals *i* and *j* in group *k* (henceforth ‘dyad *ijk*’) were recorded as being in close proximity on scan *l* on day *m* (1) or not (0). Scans for which both members of a dyad were out of sight of the experimenter were treated as missing data. In contrast, if only one member of the dyad was out of sight, then we know that the dyad was not in proximity, so such scans were assigned $t_{ijklm}=0$. We modelled this using a logit link function and Bernoulli error structure. Thus, the probability that dyad *ijk* was in proximity for a given scan $P\left( t_{ijklm}=1 \right)$ was modelled as follows:

$\frac{P\left( t_{ijklm}=1 \right)}{1-P\left( t_{ijklm}=1 \right)}=exp\left( B_{ijklm}+\beta_{last}t_{ijk(l-1)m} \right)$ Eqn. S1a

Or equivalently:

$logit\left( P\left( t_{ijklm}=1 \right) \right)=B_{ijk}+\beta_{last}t_{ijk(l-1)m}$ Eqn. S1b

Where $t_{ijk(l-1)m}$ indicates whether dyad *ijk* was in proximity during the previous scan *(l-1)*, set to zero for the first scan of each day. Therefore $\beta_{last}$ is a parameter allowing for any tendency for dyads to engage in bouts of proximity that continue across scans within a day, and thus allows for the resulting non-independence of successive scans. $B_{ijk}$ is then a linear predictor of random effects measuring the propensity for members of dyad *ijk* to be in proximity, defined as:

$B_{ijk}=a_{k}+b_{ik}+b_{jk}+c_{ijk}$ Eqn. S2

Where $a_{k}$ is a random effect of ‘group’, distributed as $a_{k}\sim N\left( \alpha,{\sigma_{group}}^{2} \right)$, $b_{ik}$ is a random effect of ‘individual’, distributed as $b_{ik}\sim N\left( 0,{\sigma_{ind}}^{2} \right)$, and $c_{ijk}$ is a random effect of ‘dyad’, distributed as $c_{ijk}\sim N\left( 0,{\sigma_{dyad}}^{2} \right)$. The parameter $\alpha$ estimates the overall population mean (on the log odds scale). Variation between dyads in the tendency to be in proximity is broken down into three sources of variation: group level, individual level, and dyad level, with the relative magnitude of each being estimated by the standard deviation (SD) of each effect, respectively $\sigma_{group}$, $\sigma_{ind}$,$\sigma_{dyad}$. The estimated relative magnitude of these effects tells us the extent to which groups vary in their propensity to be in proximity relative to the variation within groups. The overall SD within groups can be calculated as $\sigma_{within}=\sqrt{{\sigma_{dyad}}^{2}+2{\sigma_{ind}}^{2}}$, and we can then quantify the ratio of SD between/within groups as ${\sigma_{group}}/{\sigma_{within}}$. Furthermore, ${\sigma_{dyad}}/{\sqrt{2{\sigma_{ind}}^{2}}}$ provides a measurement of how dyads vary within groups. $\sqrt{2{\sigma_{ind}}^{2}}$ is used instead of $\sigma_{ind}$ since this component of variance is counted twice: via $b_{ik}$ and via $b_{jk}$. We denote this measure ${\sigma_{dyad}}/{\sigma_{IND}}$ where $\sigma_{IND}$ is the standard deviation of the variance component accounted for by individual tendencies. If, at one extreme, bonobos vary in the extent to which they are in proximity to others, but each have no preference in who they are in proximity with, we would expect$\sigma_{IND}$to be large relative to $\sigma_{dyad}$. If on the other hand bonobos differ in their preferences, we would expect $\sigma_{dyad}$ to be large relative to $\sigma_{IND}$. We also derived posterior samples for the proportion of variance explained by each component, where the total variance = ${\sigma_{group}}^{2}+{\sigma_{dyad}}^{2}+2{\sigma_{ind}}^{2}$.

Being in proximity is non-directional: if *i* was in proximity of *j* during scan *l* on day *m*, then by logical necessity *j* was also in proximity to *i*, i.e., $t_{ijklm}=t_{jiklm}$. Mathematically this is represented in the model as the constraint $P\left( t_{ijklm}=1 \right)=P\left( t_{jiklm}=1 \right)$. Practically, this was accomplished in the JAGS code by ensuring that each dyad was only counted once per scan when sampling the dependent variable. Furthermore, since the interest is social behaviour, self-proximity was not recorded, i.e., all $t_{iiklm}=$0, so we constrained $P\left( t_{iiklm}=1 \right)=0$. In the JAGS code this is implemented by ignoring the diagonal data for each scan $t_{..klm}$ when sampling the dependent variable.

Bayesian estimation was accomplished using Markov Chain Monte Carlo (MCMC) methods using the JAGS sampler^1^, via the runjags^2^ and coda packages^3^ in the R statistical environment. Vague (uninformative) priors were specified for all model parameters, with $\alpha,\beta_{last}\sim N(0,10000)$ and $\sigma_{group}$, $\sigma_{ind}$,$\sigma_{dyad}\sim U(0,10)$. We ran 1000 adaptive iterations followed by a burn-in period of 1000 iterations which was found to be sufficient for convergence of chains. We ran 20 chains in parallel on separate computer cores, sampling the MCMC every 10 iterations, running enough to obtain a thinned sample of 30,000 (taking 22 hours total). We sampled the values of the model parameters $\alpha,\beta_{last},\sigma_{group}$, $\sigma_{ind}$ and$\sigma_{dyad}$, allowing us to later derive posterior samples for ${\sigma_{group}}/{\sigma_{within}}$ and ${\sigma_{dyad}}/{\sigma_{ind}}$. We also sampled the values of $a_{k}$(each group’s coefficient) and $B_{ijk}$ enabling us to derive posterior samples for the network connection for each dyad, as well as for node-based metrics at the individual and group level (see section S2 below).

***S1.2 Distant proximity model***

The distant proximity model was identical in structure to the close proximity model described in S1.1, except $t_{ijklm}$ was replaced with $n_{ijklm}$ representing whether dyad *ijk* was recorded as either in close proximity or the same party (co-residence) on scan *l* on day *m* (1) or otherwise (0). MCMC sampling took 22 hours on 20 cores for a thinned sample of 30,000.

***S1.3 Play model***

The play model was identical in structure to the proximity model described in S1.1, except $t_{ijklm}$ was replaced with $p_{ijklm}$ representing whether dyad *ijk* was recorded as playing together on scan *l* on day *m* (1) or not (0). However, the play data also included the juveniles of the group as potential play partners but not as subjects. Thus, if an adult was engaged in play with a juvenile then it was recorded, whereas if juveniles played with one another, it was not recorded. To reflect this, the play model differed from the proximity model in the range of *j* but not in the range of *i*. As with the proximity model, *i* could take the values (1,2,… N_adult,k_), where N_adult,k_ is the number of adults in group *k*, whereas *j* could take the values (1,2,… N_adult,k_, N_adult,k_+1, N_adult,k_+2, … N_adult,k_+ N_juv,k_) where N_juv,k_ is the number of juveniles in group *k*. MCMC sampling took 61 hours on 10 cores for a thinned sample of 10,000 (autocorrelation was lower in the play posterior samples meaning a smaller posterior sample was required).

***S1.4 Groom model***

The groom model was similar in structure to the proximity model described in S1.1. However, grooming is a directional interaction: one individual was recorded as the groomer and the other as the groomed (recipient). Therefore, the dependent variable $g_{ijklm}$ represents whether *i* was recorded as grooming *j* for time period *l* on day *m*, whereas $g_{jiklm}$ represents whether *j* was recorded as grooming *i* for time period *l* on day *m*. For this reason, $P\left( g_{ijklm}=1 \right)\neq P\left( g_{jiklm}=1 \right)$ was unconstrained, and in the JAGS code the data from each dyad was counted in both directions when sampling the dependent variable. The diagonal elements were still ignored since only social grooming was of interest (self-grooming events were not included), i.e., $g_{iiklm}=$0 and $P\left( g_{iiklm}=1 \right)=0$. As with the play model, juveniles were included as potential partners but not as subjects, so again, while *i* could only take the values (1,2,… N_adult,k_), *j* could take the values (1,2,… N_adult,k_, N_adult,k_+1, N_adult,k_+2, … N_adult,k_+ N_juv,k_).

Furthermore, there was an additional random effect for grooming:

$B_{ijk}=a_{k}+b_{ik}+d_{jk}+c_{ijk}$ Eqn. S3

Here, $b_{ik}$ is now individual *i*’s propensity to groom and $d_{jk}$ is individual j’s propensity to be groomed, with standard deviation $\sigma_{rec}$ (rec= recipient), with prior $\sigma_{rec}\sim U(0,10)$. MCMC sampling took 75 hours on 20 cores for a thinned sample of 10,000 (autocorrelation was lower in the grooming posterior samples meaning a smaller posterior sample was required).

Note the following calculations due to the directional nature of grooming: $\sigma_{within}=\sqrt{{\sigma_{dyad}}^{2}+{\sigma_{ind}}^{2}+{\sigma_{rec}}^{2}}$ , $\sigma_{IND}=\sqrt{{\sigma_{ind}}^{2}+{\sigma_{rec}}^{2}}$ and total variance = ${\sigma_{group}}^{2}+{\sigma_{dyad}}^{2}+{\sigma_{ind}}^{2}+{\sigma_{rec}}^{2}$.

**S2. Posterior samples for the social networks and social network metrics**

We derived posterior distributions for the connections of each of the four networks in each group, using the posterior distributions sampled in S1 above. This has a number of advantages over calculating a single number from the raw data using e.g., the simple ratio index (SRI). First, it controls for autocorrelation in successive scans. Second, it provides a measure of uncertainty in the value of each connection. Third, this uncertainty in connection value also creates uncertainty in metrics derived from them. This resulting uncertainty can be calculated at the individual and group level, simply by calculating metrics for each iteration of the MCMC and obtaining a posterior sample, as detailed below. This in turn enables us to assess the evidence for differences between groups in network metrics.

We describe this procedure primarily in the context of the proximity model. The procedure was identical for the distant proximity model. There were some differences for the play and groom models which are highlighted where appropriate.

$B_{ijk}$ is a linear predictor measuring the propensity for members of dyad *ijk* to be in proximity, but measured on the log-odds scale. We wanted to convert this back to the probability scale as a more conventional measure of network connection strength. $B_{ijk}$ is more precisely the log-odds of dyad *ijk* being in proximity at a random point in time conditional on the fact that they were not in proximity in the previous scan. If we denote the condition that *ijk* were not in proximity previously as “previous=0”, we can derive the conditional probability:

$P\left( t_{ijk}=1|previous=0 \right)={exp(B_{ijk})}/\left( 1+\exp\left( B_{ijk} \right) \right)$ Eqn. S4

The log odds of dyad *ijk* being in proximity at random point in time conditional on the fact that they were in proximity previously is then:

$P\left( t_{ijk}=1|previous=1 \right)={exp(B_{ijk}+\beta_{last})}/\left( 1+\exp\left( B_{ijk}+\beta_{last} \right) \right)$ Eqn. S5

The unconditional probability that dyad *ijk* is in proximity at a random point can then be derived as follows:

$$P\left( t_{ijk}=1 \right)=P\left( t_{ijk}=1|previous=0 \right)\left( 1-P\left( t_{ijk}=1 \right) \right)+P\left( t_{ijk}=1|previous=1 \right)P\left( t_{ijk}=1 \right)$$

When substituting in from Eqn.4 and Eqn.5 and re-arranging we get:

$$n_{prox,ijk}=P\left( t_{ijk}=1 \right)=\frac{{exp(B_{ijk})}/\left( 1+\exp\left( B_{ijk} \right) \right)}{1+{exp(B_{ijk})}/\left( 1+\exp\left( B_{ijk} \right) \right)-{exp(B_{ijk}+\beta_{last})}/\left( 1+\exp\left( B_{ijk}+\beta_{last} \right) \right)}$$

Eqn. S6

Where $n_{prox,ijk}$ is the symmetrical connection for dyad *ijk* in the proximity network. $n_{prox,ijk}$ was calculated for each iteration of the MCMC allowing us to obtain a posterior sample for each network connection. Posterior samples for $n_{party,ijk}$ and $n_{play,ijk}$ were obtained in an identical manner. A posterior sample for $n_{groom,ijk}$ was calculated in the same way except the directed nature of the behaviour resulted in a non-symmetrical network.

As noted above, scans for which both members of a dyad were out of sight of the experimenter were treated as missing data, but if only one member of the dyad was out of sight, then we know that the dyad was not in proximity, so such scans were assigned $t_{ijklm}=0$. Thus, our procedure is analogous to the calculation of the simple ratio index (SRI) of association, in which only sampling periods in which both individuals are not recorded are excluded from the denominator. Thus, $n_{prox,ijk}$ can be taken as estimates of the SRI of association, with association derived as “being in proximity”, corrected for autocorrelation in successive scans. We are also able to derive highest posterior density intervals (Bayesian analogy to confidence intervals) for each connection and for all the derived individual and group level metrics. In our case, there was no reason to believe that individuals were more or less likely to be detected when they were apart than when they were together, so the SRI is an appropriate measure of association^4^.

Whilst play and grooming are types of interaction, as opposed to traditional criteria for association, the scan sampling nature of the data makes an association-type network most appropriate for the data we have available. For example, $n_{groom,ijk}$ estimates the probability that *i* will be grooming *j* in group *k* at a random point in time, as opposed to quantifying the rate at which *i* initiates grooming of *k* per unit time. In other words, grooming and play are treated as behavioural states as opposed to behavioural events^5^.

For the proximity networks for each group, for each iteration we also calculated 2 node-based metrics for each individual: 1) *strength*: total connection to others in the group = $\sum_{j} n_{prox,ijk}$; and 2) cluster coefficient using Ahnert et al.’s approach^6^ (see below for more details). For each iteration, we then calculated the average strength and clustering across individuals for each group enabling us to assess whether groups differed in these measures. Thus, we obtained posterior samples for network metrics at individual and group levels enabling us to derive estimates (mean of posterior) and 95% highest posterior density (HPD) intervals. We were also able to obtain 95% HPD intervals for the difference in strength and clustering between each pair of groups and test for evidence of a difference between each pair of groups (see Tables S3-S10).

Social network metrics were obtained in the same way for the proximity networks. The play and groom networks were initially non-square since we have more potential partners than subjects (see above). The total strength was calculated for each subject from the non-square matrix. However, since cluster metrics are designed for square matrices, we derived network metrics only from the square portion of the networks involving only the subjects (i.e., excluding bonobos that were only recorded as partners). In all other regards, metrics were derived for the play networks in an analogous manner to the proximity networks. Since groom networks were non-symmetrical, we derived two node-based measures for each individual: the total out-strength (grooming others) and total in-strength (being groomed). However, at the group level, only a single average strength was required since group average strength will be equal regardless of whether in- or out-strength is used. We also needed to adjust the clustering coefficient to consider the directed nature of grooming (see below).

*S2.1. Anhert et al.’s ensemble approach to clustering coefficient*

For binary unweighted networks, the local clustering coefficient for a node *i* is calculated as the number of triangles in the network including *i* divided by the number of pairs of links that include *i* (the “potential” triangles). This quantifies the extent to which *i*’s neighbours also tend to be linked to one another. Various extensions have been proposed to take into account the strength of connections in a weighted network, however, many of these do not work well for a fully connected network- where, as in our networks, all nodes are connected to all other nodes by a connection of some strength >0. Anhert et al.’s ensemble approach^6^ is valid if each connection can be thought of as a probability that a binary connection exists in one of an underlying ensemble of networks. This is the case for all our networks, in which each weighted connection is a probability that a particular interaction or association is occurring at a random moment in time. The clustering coefficient then becomes the expected number of triangles divided by the expected number of potential triangles, calculated as follows:

$$c_{i}^{e}=\frac{\sum_{j,k} p_{ij}p_{ik}p_{jk}}{\sum_{j,k} p_{ij}p_{ik}}$$

Eqn. S7

(here *i, j* and *k* are subscripts representing individuals, *k* does not represent group). To extend this logic to directed networks we simply have to allow for the fact that for every pair of outgoing connections there are two potential triangles that can be completed. Therefore, for the grooming network we used:

$$c_{i}^{e}=\frac{\sum_{j,k} p_{ij}p_{ik}p_{jk}+p_{ij}p_{ik}p_{kj}}{2\sum_{j,k} p_{ij}p_{ik}}$$

Eqn. S8

Since we are not aware of any such extensions of global clustering coefficient to fully-connected weighted networks, we used the average of $c_{i}^{e}$ across each group as a measure of the extent of clustering within each group.

**S3. Variables influencing association and interaction between dyads**

We found evidence that groups differed in the average rate of association (close proximity, distant proximity) and interaction (play and grooming) and that the variation within groups was primarily at the dyad, rather than the individual level. We next wanted to assess what variables might drive such differences between and within groups. To this end, we extended the models described in section S1 to include individual-level variables (age, sex), dyad level variables (maternal kinship, paternal kinship) and group level variables (group size, sex ratio).

Since dyad-level variation was equal to (play) or exceeded individual-level variation (close proximity, distant proximity, grooming) (see Section S4 below), this suggests that what counts is the relationship between members of each dyad with regards to each variable, e.g., same sex versus different sex. Consequently, the individual-level variables were converted into dyad-level relationships, as detailed below.

The models for close proximity, distant proximity and play were extended as follows:

$$B_{ijk}=a_{k}+b_{ik}+b_{jk}+c_{ijk}+\beta_{age}\left| {age}_{ik}-{age}_{jk} \right|+\beta_{FF}\left( {1-sex}_{ik} \right)\left( {1-sex}_{jk} \right)+\beta_{MM}{sex}_{ik}{sex}_{jk}+\beta_{matKin}{matKin}_{ijk}+\beta_{patKin}{patKin}_{ijk}$$

Eqn. S9

Where $\left| {age}_{ik}-{age}_{jk} \right|$ is the magnitude of difference in age (standardized across all groups) for dyad *ijk*, and $\beta_{age}$ estimates the effect of age difference. ${sex}_{ik}$ is the sex of individual *i* in group *k* (female=0, male=1), so $\beta_{FF}$ and $\beta_{MM}$ estimate the difference in interaction for female and male dyads respectively relative to interactions in different sex dyads. ${matKin}_{ijk}$ and ${patKin}_{ijk}$ indicates whether dyad *ijk* is maternal kin or paternal kin respectively, with $\beta_{matKin}$ and $\beta_{patKin}$ estimating the difference from dyads that are non-kin.

Since grooming is directional the extension is slightly different:

$$B_{ijk}=a_{k}+b_{ik}+d_{jk}+c_{ijk}+\beta_{age}\left( {age}_{ik}-{age}_{jk} \right)+\beta_{FF}\left( {1-sex}_{ik} \right)\left( {1-sex}_{jk} \right)+\beta_{MM}{sex}_{ik}{sex}_{jk}+\beta_{FM}\left( {1-sex}_{ik} \right){sex}_{jk}+\beta_{matKin}{matKin}_{ijk}+\beta_{patKin}{patKin}_{ijk}$$

Eqn. S10

$\beta_{age}$ is now the effect of absolute difference in age between groomer and groomed, and we have an additional effect $\beta_{FM}$ for the rate of females grooming males in mixed dyads, with the rate at which males groom females in mixed dyads set to the reference level.

We also extended the model to allow for differences between groups in group-level variables, by setting:

$\begin{matrix} a_{k}={\beta_{GS}groupsize}_{k}+{\beta_{SR}sexratio}_{k}+\epsilon_{k} \\ \epsilon_{k}\sim N\left( \alpha,{\sigma_{group}}^{2} \right) \end{matrix}$ Eqn. S11

Where ${groupsize}_{k}$ is standardized group size, and ${sexratio}_{k}$ is standardized sex ratio (females/males). Note that an effect of sex ratio is already implied by the relative effects of $\beta_{FF}$ and $\beta_{MM}$: e.g., if $\beta_{FF}$ is positive and $\beta_{MM}$ is negative then it implies that groups with a high female:male sex ratio will tend to interact more as a result of the dyad level effects. A positive effect of $\beta_{SR}$ would imply a subtly different effect- that all individuals in a group with high ${sexratio}_{k}$ tend to interact more regardless of their own sex. However, in practice we found that the data contained very little information about $\beta_{GS}$ and $\beta_{SR}$, with poor mixing of chains and posterior samples for these parameters having a very wide variance, with 0 well within the 95% HPD intervals (this is not surprising given there are only 6 groups). In other words, the effects of group size and sex ratio on our sociality measures could not be estimated with any reasonable level of certainty due to low power at the group level. Consequently, we report only the results for the effects of the individual/dyad level variables.

All $\beta$ variables were assigned the vague prior $\beta_{-}\sim N\left( 0,10000 \right)$. The MCMC was run as described in S1, with recording of the values of all $\alpha, \beta$ and $\sigma$ parameters. As before, we obtained thinned (every 10 iterations) posterior samples of 30,000 for close proximity (4 cores x 132 hours) and distant proximity (10 cores x 51 hours) and 10,000 for play (20 cores x 33 hours) and groom (16 cores x 93 hours).

We obtained the mean of the posterior sample to give an estimate for each parameter, and 95% highest posterior density intervals (HPDIs) to give plausible ranges of values. We also obtained means and 95% HPDIs for the difference in parameters between groups. HPDIs give the range of values that are most likely for a parameter or difference between two parameters, given the observed data, and are a Bayesian analogue of confidence intervals.

**S4. Results: between and within group variation**

We studied 70 bonobos across 6 independent groups (Table S1) to investigate whether sociality (operationalized by four social behaviours) in bonobos is a group-specific rather than a species-specific phenotype.

In all four analyses, the group level SD ($\sigma_{group}$) is estimated with 95% HPD intervals clearly away from zero (see Table 2 in *Main text*), indicating that there is strong evidence of systematic differences between groups in sociality that is not fully accounted by sampling error at the level of individuals and dyads. The results of the GLMM described in section S1 are presented in Table 2 (*main text*) and Table S2 (the estimated population mean for each behaviour on the log-odds scale ($\alpha$), along with estimates of the autocorrelation parameter $\beta_{last}$).

**Table S2**. Mean of posterior sample for mean and autocorrelation in all four analyses. 95% highest posterior density intervals (HPDIs) are given in brackets.

|  | Close proximity | Distant proximity | Play | Grooming |
| --- | --- | --- | --- | --- |
| Mean ($\alpha)$ | -4.84  (-5.71, -4.00) | -3.44  (-4.43, -2.41) | -9.014  (-10.80, -7.41) | -6.56  (-7.28, -5.82) |
| Autocorrelation parameter $(\beta_{last})$ | 2.58  (2.49,2.67) | 2.02  (1.97,2.07) | 1.54  (1.17,1.88) | 2.23  (2.13,2.33) |

Furthermore, the social network measures *strength* and *clustering* vary substantially between groups, such that for all four behavioural measures (close proximity, distant proximity, play and grooming) most groups differ from each other (see Tables S3-S10; Figure 2 in *main text*).

**Table S3**. Group comparisons for mean strength of *close* *proximity* connections. A shaded box indicates that the 95% Highest Posterior Density Interval for the difference between groups does not include zero (blue = row lower, orange = row higher).

**Table S4**. Group comparisons for mean clustering of *close* *proximity* connections. A shaded box indicates that the 95% Highest Posterior Density Interval for the difference between groups does not include zero (blue = row lower, orange = row higher).

**Table S5**. Group comparisons for mean strength of *distant proximity* connections. A shaded box indicates that the 95% Highest Posterior Density Interval for the difference between groups does not include zero (blue = row lower, orange = row higher).

**Table S6**. Group comparisons for mean clustering of *distant proximity* connections. A shaded box indicates that the 95% Highest Posterior Density Interval for the difference between groups does not include zero (blue = row lower, orange = row higher).

**Table S7**. Group comparisons for mean strength of *play* connections. A shaded box indicates that the 95% Highest Posterior Density Interval for the difference between groups does not include zero (blue = row lower, orange = row higher).

**Table S8**. Group comparisons for mean clustering of *play* connections. A shaded box indicates that the 95% Highest Posterior Density Interval for the difference between groups does not include zero (blue = row lower, orange = row higher).

**Table S9**. Group comparisons for mean strength of *grooming* connections. A shaded box indicates that the 95% Highest Posterior Density Interval for the difference between groups does not include zero (blue = row lower, orange = row higher).

**Table S10**. Group comparisons for mean clustering of *grooming* connections. A shaded box indicates that the 95% Highest Posterior Density Interval for the difference between groups does not include zero (blue = row lower, orange = row higher).

**S5. Results: effect of dyad-level variables**

In the main text, we present the results in written form. Here, the results for the fixed effects of the GLMM described in section S3 are presented in Table S11 and S12.

**Table S11**. Means of posterior sample for fixed effects in close proximity, distant proximity, and play analyses. 95% highest posterior density intervals (HPDIs) are given in brackets. Cells shaded grey indicate that the HPDI does not include zero and there is strong evidence of an effect. Results for the grooming analysis are given in Table S11 since some parameters or their interpretation are different due to the directional nature of grooming.

| Parameter | Description | Close proximity | Distant proximity | Play |
| --- | --- | --- | --- | --- |
| $\alpha$ | Intercept  log(odds for unrelated mixed sex dyad of same age) | -4.84  (-6.55, -3.21) | -3.52  (-4.80, -2.22) | -8.84  (-11.97, -4.97) |
| $\beta_{last}$ | Autocorrelation parameter  log(odds(last=1)/odds(last=0)) | 2.58  (2.49,2.66) | 2.02  (1.97,2.07) | 1.53  (1.19,1.88) |
| $\beta_{matKin}$ | Effect of maternal kinship  log(odds(kin)/odds(non-kin)) | 1.81  (1.00,2.66) | 1.26  (0.47,2.05) | 2.50  (1.63,3.45) |
| $\beta_{patKin}$ | Effect of paternal kinship  log(odds(kin)/odds(non-kin)) | -0.14  (-1.38,1.07) | 0.42  (-0.72,1.57) | 1.49  (0.13,2.83) |
| $\beta_{FF}$ | Effect of female dyad  log(odds(female)/odds(mixed)) | 0.70  (0.29,1.17) | 0.61  (0.16,1.09) | -0.71  (-1.93,0.49) |
| $\beta_{MM}$ | Effect of male dyad  log(odds(male)/odds(mixed)) | -0.36  (-1.18,0.46) | -0.42  (-1.20,0.33) | -0.15  (-1.42,1.19) |
| $\beta_{FF}$-$\beta_{MM}$ | log(odds(female)/odds(male)) | 1.06  (0.19,1.93) | 1.03  (0.17,1.88) | -0.56  (-2.67,1.59) |
| $\beta_{age}$ | Effect of age  change in log odds for 1 SD increase in magnitude of age difference of dyad | -0.29  (-0.54, -0.02) | -0.14  (-0.39,0.09) | -0.20  (-0.77,0.40) |

**Table S12**. Means of posterior sample for fixed effects in grooming analyses. 95% highest posterior density intervals (HPDIs) are given in brackets. Cells shaded grey indicate that the HPDI does not include zero and there is strong evidence of an effect.

| Parameter | Description | Grooming |
| --- | --- | --- |
| $\alpha$ | Intercept  log(odds for male grooming an unrelated female of same age) | -6.91  (-8.92, -5.05) |
| $\beta_{last}$ | Autocorrelation parameter  log(odds(last=1)/odds(last=0)) | 2.23  (2.13,2.32) |
| $\beta_{matKin}$ | Effect of maternal kinship  log(odds(kin)/odds(non-kin)) | 2.76  (2.06,3.44) |
| $\beta_{patKin}$ | Effect of paternal kinship  log(odds(kin)/odds(non-kin)) | -0.36  (-1.45,0.65) |
| $\beta_{FF}$ | Effect of female dyad  log(odds(female dyad)/odds(male grooming female)) | 0.29  (-1.75,2.21) |
| $\beta_{MM}$ | Effect of male dyad  log(odds(male dyad)/odds(male grooming female)) | -0.53  (-1.89,0.80) |
| $\beta_{FM}$ | Effect of female grooming male  log(odds(female grooming male)/odds(male grooming female)) | -0.07  (-2.42,2.31) |
| $\beta_{FF}$-$\beta_{MM}$ | log(odds(female dyad)/odds(male dyad)) | 0.82  (-1.09,2.77) |
| $\beta_{age}$ | Effect of age  change in log odds for 1 SD increase in difference in age of groomer and groomed | -0.20  (-0.85,0.40) |

**S6. Results: variation in clique size**

The models described and reported above assume that each dyad interacts independently in time, i.e., the status of dyad *ij* at scan *t* (interacting or not) is independent of the status of all other dyads *kl*, *ik*, or *kj* at scan *t*. This is likely to be a reasonable simplification and approximation for establishing differences in overall dyad-level rates of interaction between groups (section S4) and testing for the effect of dyad-level variables on rates of interaction (section S5). However, we conjectured that groups might also differ in the extent to which they formed cliques of interacting individuals^7,8^ (e.g., more than 2 individuals at the same time), over and above that predicted by the dyadic interaction rates estimated in the model. Given that such cliques are exponents of the social behaviour we are interested in for the assessment of intraspecific variation in sociality, we developed the following analytical approach to test for group differences in clique size for all four social measures.

For a given matrix of dyadic interaction probabilities, one can generate a simulated distribution of clique sizes for each individual in a group (for each interaction type) with the number of scans matching that in the real data. By doing this for each point in the posterior sample, generated from the models described in S1, we can obtain a posterior distribution for the predicted distribution of clique sizes predicted by the model. For example, the results for “close proximity” for one of the groups (i.e., Apenheul) are shown in Fig. S1 below.


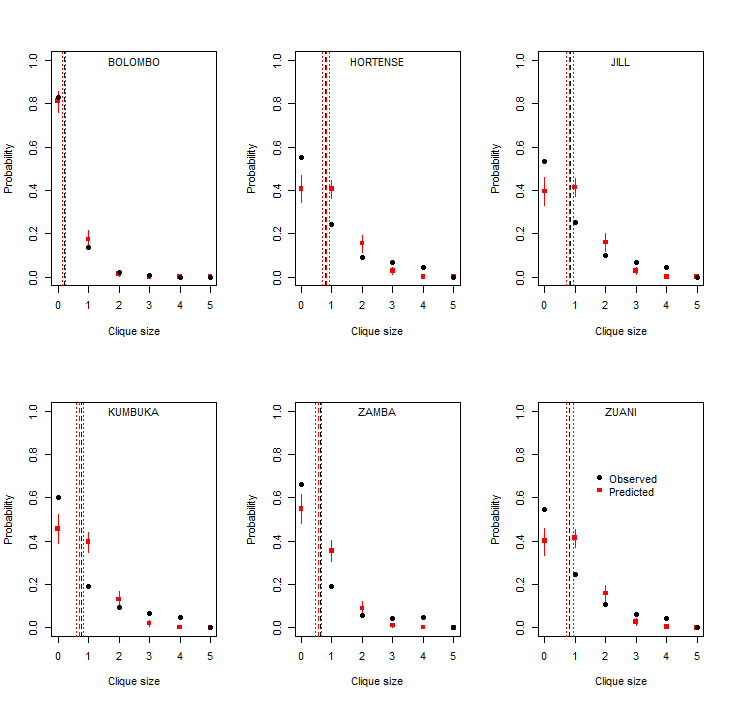


**Figure S1.** The predicted and observed clique size distributions for *close* *proximity* for individuals at Apenheul. “Clique size” refers to the number of other individuals in proximity to the focal individual at a random point in time. Red points give the probability predicted by the model, with error bars showing the 95% HPDI. Black points give the observed distribution of clique sizes for that individual. The vertical dashed black line shows the observed mean clique size for each individual, the vertical dashed red line shows the predicted mean clique size (visible only when it is not overlaid exactly by the observed mean), with dotted lines showing the 95% HPDI.

Notably, the model predicts the mean clique size well for each individual. However, there is a systematic difference between the predicted and observed clique size distributions. In general, larger cliques were observed more often than would be expected by the model, whereas small (1-2) and zero cliques were less common than expected by the model. Note that these two patterns are logically linked- since the mean clique size is predicted well, more large cliques than predicted necessarily means fewer small cliques than predicted. Similar patterns were observed for other groups and behaviour patterns (except grooming).

We found that mean clique size is predicted well across all individuals, groups, and interaction types. Consequently, the tendency to form large cliques more often than expected by the model is represented by the degree to which the variance in observed clique size is greater than the variance in clique size predicted by the model. We calculated the observed variance in clique size at the group level by averaging the clique size distribution across all individuals in the group and calculating the variance from the resulting discrete probability distribution. We repeated this for each point of the posterior distribution to obtain a posterior sample for the expected variance in clique size. We divided the former by the latter to obtain a posterior sample for the ratio of observed/predicted variance in clique size. This allowed us to obtain an estimate (mean of posterior) for each group with 95% HPDI, and calculate the posterior probability that each pair of groups differed in this measure.

*Distant proximity*. All groups formed large *party* cliques more often than expected by the dyadic interaction rates alone. There is also strong evidence of differences between the groups in the extent to which they did this (Figure S2).

**Figure S2.** The ratio of observed/predicted variance in *distant proximity* clique size for each group. The mean of the posterior sample is plotted with 95% HPDI. The dashed line at y=1 shows the ratio for if the observed distributions matched those predicted by the model. Dashed lines at the top of the plot link groups that did *not* differ significantly (posterior probability <95%), e.g., there is not strong evidence that Apenheul differs from Twycross, nor that Twycross differs from Planckendael, but there is strong evidence that Apenheul differs from Planckendael.

*Close proximity*. In contrast, only two groups (Apenheul and Stuttgart) formed large proximity cliques more often than predicted by the dyadic interaction rates alone. But there is still strong evidence of a difference between groups, with Wuppertal even forming large cliques less often than predicted by the dyadic interaction rates (Figure S3). Notably, for both criteria representing social closeness (i.e., close proximity and distant proximity), Apenheul shows the strongest tendency to from large cliques and Wuppertal the smallest tendency.

**Figure S3.** The ratio of observed/predicted variance in *close* *proximity* clique size for each group. The mean of the posterior sample is plotted with 95% HPDI. The dashed line shows the ratio if the observed distributions matched those predicted by the model. Dashed lines at the top of the plot link groups that did not differ significantly (posterior probability <95%).

*Social play*. There was evidence that three groups (Frankfurt, Apenheul and Stuttgart) formed large play cliques more often than expected by dyadic interaction rates alone. There was some evidence of differences between the groups, but the group ranking is less well resolved than for the close and distant proximity analyses, due to the rarer nature of play interactions across all groups (Figure S4).

**Figure S4.** The ratio of observed/predicted variance in *play* clique size for each group. The mean of the posterior sample is plotted with 95% HPDI. The dashed line shows the ratio if the observed distributions matched those predicted by the model. Dashed lines at the top of the plot link groups that did not differ significantly (posterior probability <95%).

*Grooming*. Finally, there was no evidence that any groups tended to form larger grooming cliques more often than expected by the model, with the mean of the posterior distribution being slightly less than 1 for all groups and the 95% HPDIs for all groups included 1, suggesting the grooming model predicts clique size distribution very well.

Furthermore, there is no evidence of differences in deviation from the model predictions between groups. This reflects that within a single scan an individual would almost always only ever groom one other individual at most, and this fit well with the predictions of the grooming model (Figure S5).

**Figure S5.** The ratio of observed/predicted variance in *grooming* clique size for each group. The mean of the posterior sample is plotted with 95% HPDI. The dashed line shows the ratio if the observed distributions matched those predicted by the model. Dashed lines at the top of the plot link groups that did not differ significantly (posterior probability <95%).

**Appendix I *Subject information***

**Table S1.** Demographic details of the bonobos, including sex, age (in years), and zoological institution.

| Subject | Sex | Age | Zoo |
| --- | --- | --- | --- |
| Bolombo | Male | 14 | Apenheul |
| Hongo | Male | 6 | Apenheul |
| Hortense | Female | 34 | Apenheul |
| Jill | Female | 27 | Apenheul |
| Kumbuka | Female | 13 | Apenheul |
| Makasi | Male | 3 | Apenheul |
| Monyama | Female | 2 | Apenheul |
| Yahimba | Female | 2 | Apenheul |
| Zamba | Male | 14 | Apenheul |
| Zuani | Female | 22 | Apenheul |
| Bashira | Female | 8 | Frankfurt |
| Bili | Male | 5 | Frankfurt |
| Heri | Male | 13 | Frankfurt |
| Kamiti | Female | 27 | Frankfurt |
| Kutu | Female | 16 | Frankfurt |
| Ludwig | Male | 29 | Frankfurt |
| Margrit | Female | 63 | Frankfurt |
| Mixi | Female | 12 | Frankfurt |
| Natalie | Female | 50 | Frankfurt |
| Nyota | Male | 7 | Frankfurt |
| Omanga | Female | 5 | Frankfurt |
| Pangi | Female | 5 | Frankfurt |
| Panisco | Male | 4 | Frankfurt |
| Sambo | Male | 2 | Frankfurt |
| Tikala | Female | 1 | Frankfurt |
| Zomi | Female | 16 | Frankfurt |
| Djanoa | Female | 18 | Planckendael |
| Habari | Male | 6 | Planckendael |
| Lina | Female | 27 | Planckendael |
| Lingoye | Female | 5 | Planckendael |
| Louisoko | Male | 15 | Planckendael |
| Lucuma | Male | 10 | Planckendael |
| Nayoki | Female | 1 | Planckendael |
| Vifijo | Male | 18 | Planckendael |
| Banbo | Female | 12 | Stuttgart |
| Chipita | Female | 21 | Stuttgart |
| Haiba | Female | 13 | Stuttgart |
| Hermien | Female | 36 | Stuttgart |
| Huenda | Female | 8 | Stuttgart |
| Kasai | Male | 10 | Stuttgart |
| Kianga | Female | 8 | Stuttgart |
| Kombote | Female | 47 | Stuttgart |
| Liboso | Female | 17 | Stuttgart |
| Lubao | Male | 0,1 | Stuttgart |
| Mobikisi | Male | 34 | Stuttgart |
| Nayembi | Female | 8 | Stuttgart |
| Ximba | Female | 17 | Stuttgart |
| Zorba | Male | 33 | Stuttgart |
| Banya | Female | 24 | Twycross |
| Cheka | Female | 17 | Twycross |
| Diatou | Female | 36 | Twycross |
| Gemena | Female | 7 | Twycross |
| Kakowet | Male | 33 | Twycross |
| Keke | Male | 20 | Twycross |
| Kichele | Female | 24 | Twycross |
| Lopori | Female | 2 | Twycross |
| Luo | Male | 11 | Twycross |
| Malaika | Female | 3 | Twycross |
| Maringa | Female | 15 | Twycross |
| Mokonzi | Male | 0,4 | Twycross |
| Winton | Male | 3 | Twycross |
| Ayubu | Male | 2 | Wuppertal |
| Azibo | Male | 2 | Wuppertal |
| Birogu | Male | 23 | Wuppertal |
| Busira | Female | 9 | Wuppertal |
| Eja | Female | 23 | Wuppertal |
| Lisala | Female | 32 | Wuppertal |
| Lusambo | Male | 33 | Wuppertal |
| Mato | Male | 49 | Wuppertal |
| Muhdeblu | Female | 12 | Wuppertal |

*age at the start of the study

**References**

1. Plummer, M. JAGS : A Program for Analysis of Bayesian Graphical Models Using Gibbs Sampling JAGS : Just Another Gibbs Sampler. (2003).

2. Denwood, M. J. runjags: An R package providing interface utilities, model templates, parallel computing methods and additional distributions for MCMC models in JAGS. *J. Stat. Softw.* **71**, 1–25 (2016).

3. Plummer, M., Best, N., Cowles, K. & Vines, K. {CODA}: Convergence Diagnosis and Output Analysis for {MCMC}. *R News* vol. 6 7–11 http://cran.r-project.org/doc/Rnews/ (2006).

4. Hoppitt, W. J. E. & Farine, D. R. Association indices for quantifying social relationships: how to deal with missing observations of individuals or groups. *Anim. Behav.* **136**, 227–238 (2018).

5. Martin, P. R. & Bateson, P. P. G. *Measuring behaviour: an introductory guide*. (Cambridge University Press, 2007).

6. Ahnert, S. E., Garlaschelli, D., Fink, T. M. A. & Caldarelli, G. Ensemble approach to the analysis of weighted networks. *Phys. Rev. E - Stat. Nonlinear, Soft Matter Phys.* **76**, (2007).

7. Girard-Buttoz, C. *et al.* Variable use of polyadic grooming and its effect on access to social partners in wild chimpanzees and bonobos. *Anim. Behav.* **168**, 211–224 (2020).

8. Sakamaki, T. Social grooming among wild bonobos (Pan paniscus) at Wamba in the Luo Scientific Reserve, DR Congo, with special reference to the formation of grooming gatherings. *Primates.* **54**, 349–59 (2013).
